# Calibration‐Jitter: Augmentation of hyperspectral data for improved surgical scene segmentation

**DOI:** 10.1049/htl2.12102

**Published:** 2024-11-29

**Authors:** Alfie Roddan, Tobias Czempiel, Daniel S. Elson, Stamatia Giannarou

**Affiliations:** ^1^ The Hamlyn Centre for Robotic Surgery Department of Surgery and Cancer Imperial College London London UK

**Keywords:** biomedical imaging, brain, image segmentation

## Abstract

Semantic surgical scene segmentation is crucial for accurately identifying and delineating different tissue types during surgery, enhancing outcomes and reducing complications. Hyperspectral imaging provides detailed information beyond visible color filters, offering an enhanced view of tissue characteristics. Combined with machine learning, it supports critical tumor resection decisions. Traditional augmentations fail to effectively train machine learning models on illumination and sensor sensitivity variations. Learning to handle these variations is crucial to enable models to better generalize, ultimately enhancing their reliability in deployment. In this article, *Calibration‐Jitter* is introduced, a spectral augmentation technique that leverages hyperspectral calibration variations to improve predictive performance. Evaluated on scene segmentation on a neurosurgical dataset, *Calibration‐Jitter* achieved a F1‐score of 74.35% with SegFormer, surpassing the previous best of 70.2%. This advancement addresses limitations of traditional augmentations, improving hyperspectral imaging segmentation performance.

## Graphical abstract

We present Calibration‐Jitter, a spectral augmentation technique for hyperspectral imaging that improves neurosurgical scene segmentation by addressing illumination and sensor sensitivity variations. Tested on a neurosurgical dataset, Calibration‐Jitter enhanced model performance, achieving a 74.35 F1‐score with SegFormer, surpassing previous benchmarks.

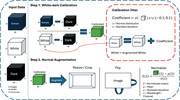

## 1 INTRODUCTION

Recently, the use of hyperspectral imaging has shown promise in surgical oncology and in particular in neurosurgery for intraoperative margin delineation [1, 2]. We leverage the information found in the array of wavelengths to create an online and label‐free diagnostic tool of the surgical scene [3].

Applying Machine Learning (ML) and Deep Learning (DL) models intraoperatively to analyse hyperspectral data for tissue characterisation is an important tool for the decision‐making processes. DL models are able to draw correlations in high dimensional spaces where statistical models often fail. Recent surgical applications using hyperspectral data have utilized DL models for various tasks, including tongue tumor detection [4], retinal vasculature segmentation [5], and neurosurgical semantic scene segmentation [6]. DL can efficiently handle the high dimensionality of the modality [7] and offers high predictive power. However this is at the cost of interpretability and bias [8]. Deep learning models act as “black boxes,” making their decision‐making process opaque. This lack of transparency can obscure errors or biases, inherited from the training data, leading to potentially unfair or unwanted predictions.

To develop computer‐aided intervention (CAI) tools, we need hyperspectral imaging (HSI) datasets comprising of spectral data from multiple and diverse surgical procedures for precise tissue characterization. Making these acquisitions comparable to each other through calibration will play a crucial role in mitigating aspects specific to the data capturing environment for HSI. Calibration addresses camera‐specific and light‐source‐specific factors, helping to standardize and enhance the comparability of HSI data in different environments and acquisition settings [9]. After calibration, the HSI data can be analyzed using statistical tools or DL approaches to predict information that aids the surgeons. These decision guidance models must be robust, interpretable and provide accurate predictors for surgeons to utilise them as CAI tools in critical environments [10]. To achieve this, DL models require large amounts of training data in order to maximise their predictive power and to minimise bias [11]. In limited data scenarios, DL models tend to over fit and start to memorize the training examples instead of learning the underlying patterns, not generalizing to unseen data. For this reason it is vital that augmentation techniques are developed to increase the volume of training data, helping to improve performance and accuracy for CAI tools.

In the RGB domain geometric, regularizing and color augmentations are routinely used to help alleviate overfitting and improve feature learning of DL models by providing more data which also encourages the models to learn representative features of the task [12, 13].

Augmentation techniques based on geometric transformations slightly perturb the images using techniques such as rotation, flipping or cropping. These techniques artificially increase the size and diversity of the training dataset which provides DL models with more examples to learn from. These small perturbations demotivate the model from simply memorizing the spatial distribution of the original images [13].

Regularization augmentations such as Cutout [14] and Cutmix [15] act to remove some image information, forcing the model to learn in more challenging scenarios [13]. Cutout randomly removes square regions from the input image, while Cutmix cuts and pastes patches from one part of an image onto another. By introducing these forms of variations, the model is encouraged to capture generalizable representations, leading to better performance and reduced overfitting. These techniques have proven effective across various computer vision tasks, including image classification, object detection, and semantic segmentation.

Color augmentations act to ensure the model is learning semantic features of objects and their texture [13]. Changes in brightness, contrast, hue and swapping of color channels help overcome lighting biases, ensuring invariance to illumination conditions and color variations. Rather than overfitting to the specific lighting and color characteristics of the training data [13], this helps the model to generalize better to different lighting scenarios, camera sensors, and imaging environments that it may encounter during deployment.

Recent work in the medical domain has suggested that augmentations should be specific to the modality of the training data [16]. While standard RGB augmentations are suitable for natural images, medical imaging modalities such as computed tomography scans, magnetic resonance imaging or ultrasound require specialized augmentation techniques tailored to their unique characteristics and artifacts to ensure effective learning and generalization. In addition, it is important to ensure that the augmentation techniques used are representative of a dataset's natural variations in order to avoid introducing bias and discourage the model from learning features that are not representative. Therefore, augmentations need to be designed and parameterised to be dataset and architecture invariant and focusing on the data modality itself.

Augmentations tailored to the hyperspectral domain in medical applications are often specifically designed for the respective task or architecture. For example, an organ cutmix method has been proposed for surgical semantic scene segmentation which moves an organ's spatial location [17]. This method improved robustness, but requires dense annotations of the surgical scene which is not always available. For lesional classification, sparse coding algorithms like K‐SVD and A+ have been proposed for augmenting hyperspectral image [18]. Sparse coding learns an overcomplete dictionary from the training data and encodes each image patch as a sparse combination of dictionary elements. By adjusting the sparse coding parameters, new hyperspectral image patches are generated and added to the original training set. While sparse coding is effective for data synthesis, it cannot realistically simulate changes in lighting, noise patterns, occlusions, or other real‐world variations that might be encountered during deployment.

Outside the medical domain, remote sensing offers some HSI specific augmentation techniques. The use of random occlusion, a data augmentation technique, to mitigate overfitting in convolutional neural networks (CNNs) for HSI classification was also introduced [19]. The augmentation randomly occludes rectangular spatial regions in the HSI during training, generating augmented images with varying levels of occlusion. As stated before the occlusion acts as a regularizer. By introducing this randomized occlusion noise during training, the model becomes less sensitive to local spatial patterns and learns to focus on features across the entire image. This type of augmentation shows promising results but other important aspects of image augmentation such as as illumination conditions, sensor noise characteristics, and geometric transformations are not considered. Additionally, superpixel patches [20], pixel‐level combinations [21] and label manipulation [22] are used to alter the ground truth label in a controlled manner to increase variation. While these methods capture local spatial structures, grouping always has the risk of overlooking pixel level characteristics.

In summary, while various data augmentation techniques have been proposed for HSI, including occlusion, superpixel transformations, and multilabel sampling, a notable limitation is their lack of brightness and color variations. Unlike RGB images, where color augmentations such as brightness, contrast, hue and swapping color channels are commonly employed, these techniques are not directly applicable to hyperspectral data. This is specifically important to surgical HSI data as they may introduce unrealistic or physically implausible variations in the spectral signatures of different tissue types. A hyperspectral augmentation targeting the surgical domain must take great care in preserving the spectral continuity correlated with the spectral fingerprint of different tissue types. This highlights the need for developing augmentation techniques tailored specifically for surgical HSI, which target changes in illumination conditions, sensor characteristics, and other factors that does not interrupt the spectral response of biological tissue.

In the operating room (OR), lighting conditions can vary significantly due to differences in OR equipment, light source manufacturers, and surgeons' personal lighting preferences. Our approach addresses this variability by offering a simple yet effective method to manage lighting differences, thus enhancing the clinical utility of HSI. While HSI has considerable potential, fully realizing it requires large datasets paired with hyperspectral‐specific augmentation techniques such as the one we propose. We believe that only the combination of both can unlock HSI's full potential, ultimately improving patient outcomes and incorporating functional information visualization, including tissue oxygenation [23], spectral fingerprint classification [24] or semantic scene segmentation [17], into clinical practice.

The contribution of this paper is threefold:

- We introduce a novel spectral augmentation technique called *Calibration‐Jitter* using variations in the calibration step.
- We evaluate the performance of *Calibration‐Jitter* across SegFormer and UNet segmentation models on a hyperspectral neurosurgical dataset and demonstrating superior performance.
- We create a simplified one‐step pipeline for sparse semantic segmentation on the hyperspectral imaging benchmark [24], outperforming the state‐of‐the‐art in terms of F1.

## 2 METHOD

### 2.1 Calibration‐Jitter

**FIGURE 1:**
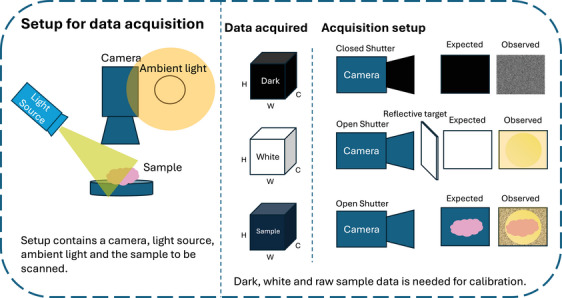
Hyperspectral data acquisition setup for surgical applications. Left: Standard data acquisition setup, consisting of a hyperspectral camera mounted on an articulated arm or robotic system, positioned above the surgical field to capture hyperspectral images of the target tissue or organ. Illumination sources can be broadband light sources or specialized hyperspectral systems. Right: Calibration setup for acquiring dark reference with the illumination source off, white reference using a standardized white reference target, and raw sample measurement. The reference measurements are used to calibrate the acquired hyperspectral data for comparable spectral analysis and tissue characterization.

Figure 1 depicts a typical hyperspectral acquisition setup, highlighting that a camera records light from the specimen but the data is also affected by factors such as light leakage and sensor noise (approximated using the dark reference) and different illumination settings (compensated using the White reference). During the surgical procedure, the hyperspectral camera acquires a three‐dimensional data cube, where two dimensions represent the spatial information (x,y), and the third dimension corresponds to the spectral information (λ), typically spanning the visible and near‐infrared wavelength ranges. Each recorded hyperspectral image is defined as $X\in R^{C\times H\times W}$ where C is the number of spectral channels, H is the height of the image and W is the width of the image. For a segmentation dataset, a hyperspectral image has a corresponding annotation of the surgical scene $y\in {\left\{0,1,\text{…},Y_{N}\right\}}^{H\times W}$ where the pixel value represents the class up to the number of classes, $Y_{N}$. The dataset is then formally denoted as $\text{Dataset}={\left\{\left(X_{i},y_{i}\right)\right\}}_{i=1}^{N}$ where N is the total number of images. The dataset consists of acquisitions taken across several cohorts and possibly locations thereby increasing the overall variability of the dataset [24].

Changes in illumination conditions and camera settings between various locations or times of the day can potentially affect the comparability of spectral measurements. Different illumination sources affect the color and intensity of the light captured. The color and brightness representation of the same surgical scene can vary due to differences in camera sensitivity and response to various wavelengths, even among cameras of the same model. For this reason, calibration is a crucial step in hyperspectral data acquisition to ensure comparable spectral measurements. The calibrated reflectance R(λ) at a given wavelength λ is calculated as:

(1) $$R\left(\lambda \right)=\frac{Raw(\lambda )-D(\lambda )}{W(\lambda )-D(\lambda )},$$

where Raw(λ) is the raw digital value recorded by the sensor, W(λ) is the white reference measurement obtained from a near‐perfect diffuse reflector (e.g. a very reflective surface), and D(λ) is the dark reference measurement obtained with the camera shutter closed or lens capped. The white reference W(λ) accounts for the non‐uniform spectral response of the sensor and the illumination source, while the dark reference D(λ) corrects for the dark current and background light leakage. After the white‐dark calibration, the reflectance values R(λ) range from 0 to 1, representing the fraction of incident light reflected by the sample at each wavelength.

Typically in a hyperspectral dataset, after calibration, the spectra are often smoothed and then normalized (min‐max) per‐pixel [24]. Spectral smoothing is applied to further refine the data by averaging out random noise and minor fluctuations. The min‐max normalization allows for illumination intensity changes [17], since spectra of a certain tissue type should match regardless of the source brightness or the distance from the camera. This process removes the absolute intensity information at each pixel, R(x,y), rendering standard color augmentation techniques such as per‐channel brightness or contrast augmentations ineffective for simulating illumination variations in the normalized hyperspectral data.

### 2.2 Geometric augmentation

Small training datasets exhibit limitations in spatial variation and the DL model may then memorize the spatial locations of certain tissues rather than learning the spectral features. Therefore, we employ geometric augmentations to artificially increase the diversity and spatial representation of tissues within the dataset. A common augmentation pipeline, which we denote as “normal”, involves geometric transformations, such as rotation, cropping and flipping, to simulate different orientations and perspectives of the surgical field.

For image X, the rotation is defined as,

(2) $$X^{rot}={\text{Rot}}_{\theta }\left(X\right),$$

where ${\text{Rot}}_{\theta }$ is the rotation operation by an angle θ, and $X^{rot}$ is the output rotated hyperspectral image. Cropping is defined as,

(3) $$X^{crop}={\text{Crop}}_{x_{1}:x_{2},y_{1}:y_{2}}\left(X\right),$$

where ${\text{Crop}}_{x_{1}:x_{2},y_{1}:y_{2}}$ is the cropping operation that extracts a region of interest defined by the pixel coordinates $\left(x_{1},y_{1}\right)$ and $\left(x_{2},y_{2}\right)$, and $X^{crop}$ is the cropped hyperspectral image. Flipping is defined as,

(4) $$X^{flip}={\text{Flip}}_{axis}\left(X\right),$$

where ${\text{Flip}}_{axis}$ is the flipping operation along a specified axis (e.g. horizontal or vertical), and $X^{flip}$ is the flipped hyperspectral image.

While the above geometric augmentations are effective in introducing spatial variations and increasing the diversity of the training data, they do not explicitly account for variations in illumination conditions and sensor sensitivities, which can significantly impact the spectral signatures of the hyperspectral images.

We aim to enhance the hyperspectral dataset by introducing variations that simulate real‐world changes in illumination and sensor noise. As previously stated, the min‐max normalization process at each pixel after calibration standardizes the intensity values across all channels (same as other normalizations such as L1). This prevents us from replicating changes in illumination and sensor noise per channel. Attempting to introduce these variations would disrupt the continuity of the spectral data, resulting in a disjointed spectrum. Therefore, to achieve illumination and sensor noise changes, we propose *Calibration‐Jitter*, a novel hyperspectral data augmentation method that adjusts the white reference data captured during the calibration step, before the non‐invertible normalization step. The white reference serves as a standardized representation of the illumination conditions during data acquisition. By introducing controlled offsets to the white reference, we can simulate different illumination scenarios and generate augmented samples that capture these variations. Specifically, we offset the white reference by a coefficient proportional to its standard deviation, ensuring that the augmented samples remain within a reasonable intensity range for each image and channel. The new augmented white reference is calculated by:

(5) $$W{\left(\lambda \right)}_{\text{augmented}}=W\left(\lambda \right)+\text{STD}\left(\lambda \right)*U\left(\text{low, high}\right),$$

where W(λ) is the original white reference measurement, STD(λ) is the standard deviation at each wavelength of the white reference, and U(low, high) is a coefficient drawn from the uniform distribution U with a lower and upper bound (denoted as low and high). The coefficient parameter controls the extent of the offset. The augmented white reference, $W{\left(\lambda \right)}_{\text{augmented}}$, is then used in Equation (1) to calibrate the raw sample measurements.

**FIGURE 2:**
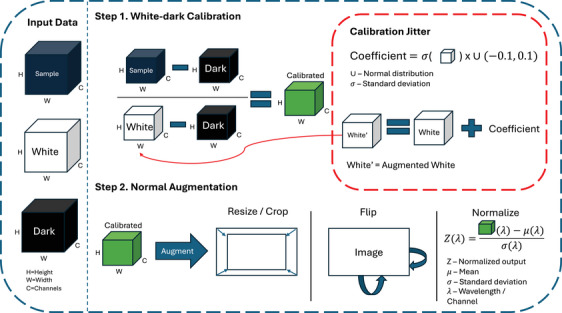
Example pipeline of dataloader. Hyperspectral data is first calibrated and then augmented. With the proposed method, we augment the white reference in step 1.

The standard pipeline of calibration is shown in Figure 2, where the proposed method is highlighted in the red box. By incorporating these augmented samples into the training process, the model is exposed to a broader range of illumination variations, enhancing its ability to learn illumination‐invariant features and generalize to scenarios with varying lighting conditions. This augmentation technique complements the normal transformations, providing a comprehensive approach to data augmentation that captures spatial, sensor and illumination variability, ultimately improving the performance and generalization capabilities of hyperspectral tissue characterization models.

## 3 RESULTS

### 3.1 Dataset

The Hyperspectral imaging benchmark (HIB) dataset [24] is comprised of a total of 61 images of 34 patients. Despite being the largest hyperspectral neurosurgical dataset to date, this benchmark is small in comparison to the datasets available in the RGB domain. This highlights the scarcity of annotated data in this field and therefore the crucial need for data augmentation techniques. We follow the same per patient data splits as reported in the benchmark paper. The included hyperspectral images have different heights and widths but all have 826 wavelengths bands, ranging from 400.5 nm to 1000.7 nm, with a sampling step size of 0.73 nm. To preprocess the raw dataset, we again follow the same processing procedure as reported in the original paper:

1 White‐dark calibration.
2 Smoothing filter across the spectral dimension (kernel size of 5).
3 Remove the first 56 and last 126 bands.
4 Resample along the channel dimension to the optimal sampling interval [25].
5 Min‐max normalization along the spectral dimension.

**FIGURE 3:**
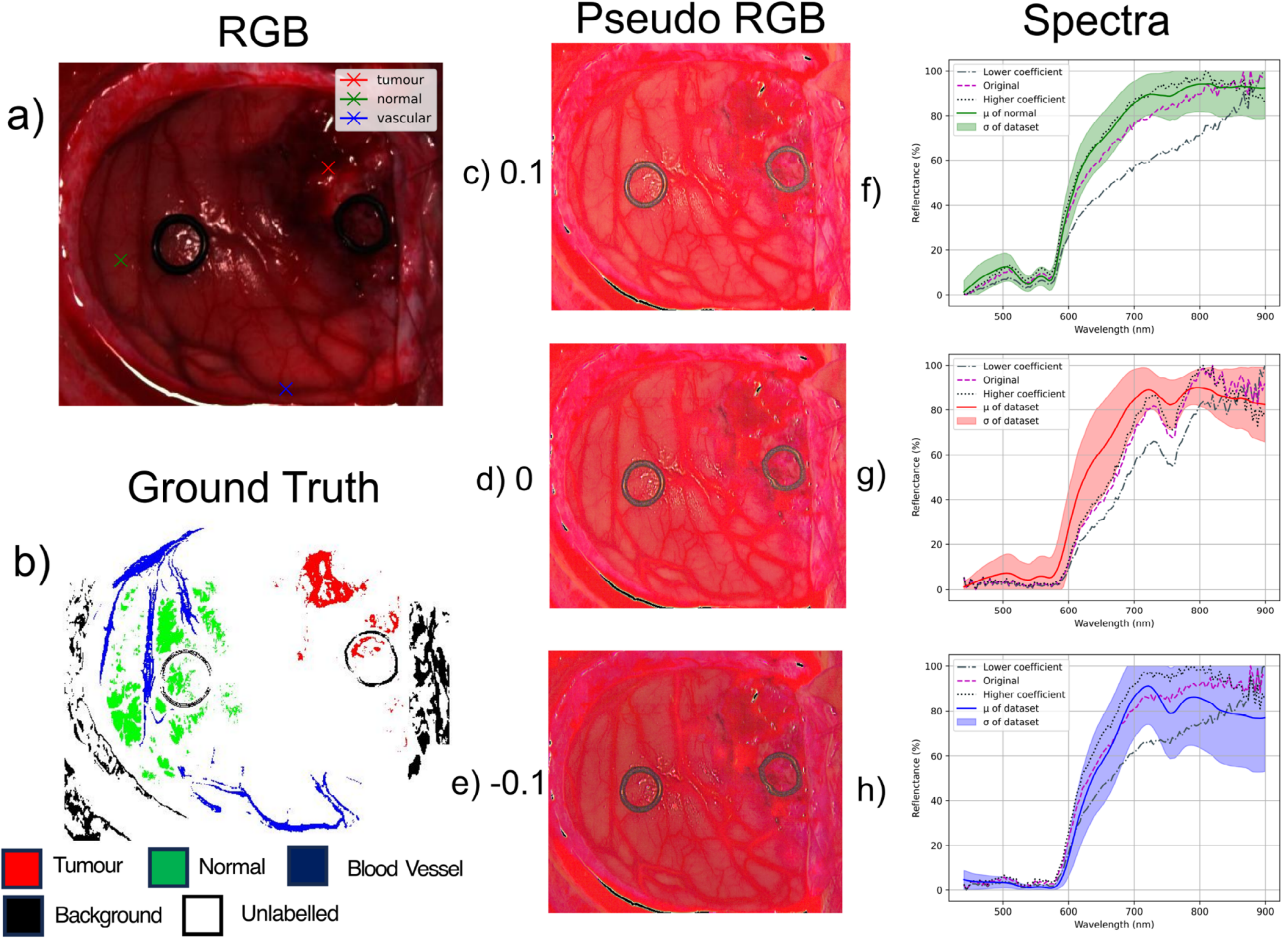
Examples of augmentation on the same image. (a) RGB image with labelled points. (b) ground truth annotation. (c) Psuedo RGB and spectra of corresponding points at labelled points for coefficient of 0.1. (d) Psuedo RGB and spectra of corresponding points at labelled points for coefficient of 0, that is, normal white‐dark calibration. (e) Psuedo RGB and spectra of corresponding points at labelled points for coefficient of −0.1. Psuedo RGB is formed by the closest available wavelengths to 630 nm (red), 540 nm (green), 480 nm (blue). (f)–(h) Higher, lower and zero coefficient (0.1, −0.1 and 0, respectively) for the corresponding class. Alongside the classes mean and standard deviation over the whole dataset. (f) Normal class, (g) tumour class and (h) blood vessel class.

**FIGURE 4:**
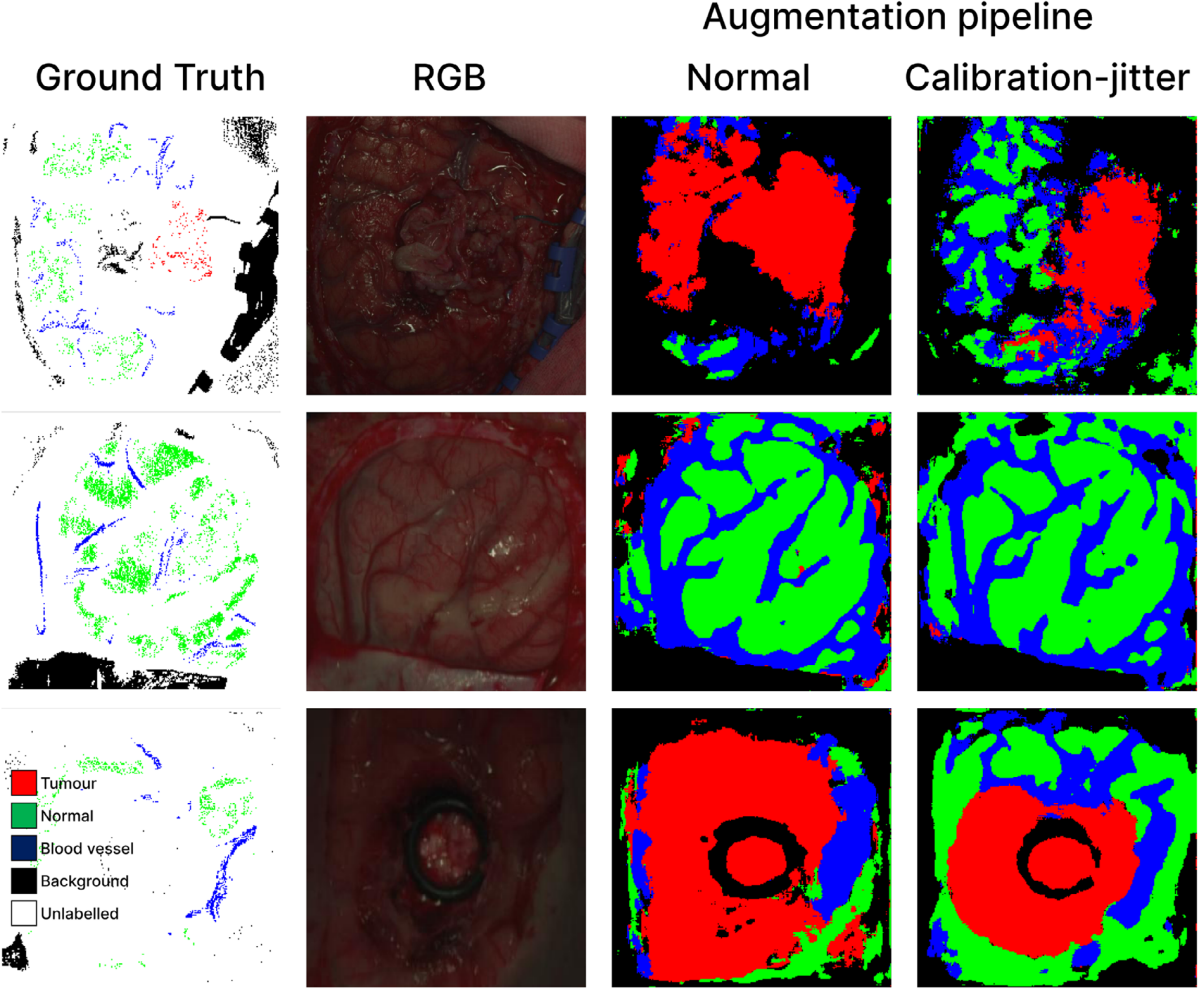
Qualitative results from SegFormer model with and without augmentation applied. First column is the ground truth annotation. Second column is the RGB image corresponding to the ground truth. Third column is normal augmentation (no *Calibration‐Jitter*) and last column is with *Calibration‐Jitter* applied. The Calibration‐Jitter images have significantly less False Positives tumor predictions and even under difficult lighting conditions the Normal and Blood vessels could still be identified as seen in row 3 Jitter.

The final number of output bands is 128, ranging between 440.45 nm and 909.9 nm. The annotations provided were crafted using a custom algorithm described in [25] and are sparse, as shown in Figures 3 and 4. Over the entire dataset, there are five categorized labels namely, unlabelled, normal, tumor, blood vessel and background with pixel label counts 11,805,660, 308,328, 37,399, 112,407 and 445,540, respectively. The unlabelled class accounts for 92.9% of all pixels whereas tumor accounts for 0.3%, therefore we classify this task as a imbalanced sparsely‐annotated semantic segmentation task.

### 3.2 Segmentation models

The benchmark paper proposes using a per‐pixel spectral classification followed by a spatial smoothing [24]. We however use a one‐step semantic segmentation pipeline, with one forward pass of a segmentation model. Treating this as a semantic segmentation task allows us to leverage the state‐of‐the‐art deep learning segmentation networks on offer. For this study we select a UNet as it is one of the most used architectures for medical imaging [26] and SegFormer a transformer based architecture recently proposed and the new SOTA for many segmentation tasks [27]. For the UNet, we use a ResNet34 backbone [28]. All models were trained with AdamW [29] with learning rate set to 1e−4, betas (0.9,0.999) and weight decay of 1e−2, with a batch size of 8. We implemented these models using PyTorch with help from external libraries for specific model implementations; SMP segmentation for UNet, hugging face for SegFormer and PyTorch lightning for the training framework [30, 31, 32, 33]. All models where trained on a single Nvidia RTX 3090 GPU. We chose the partial weighted cross‐entropy loss.

(6) $$L=-\sum_{i=1}^{Y_{N}}w_{c}\cdot y_{o,c}\cdot \log\left(p_{o,c}\right),$$

where L is the loss, $Y_{N}$ is the number of classes, $w_{c}$ is the weight assigned to class c, $y_{o,c}$ is the binary indicated for class c observation o and $p_{o,c}$ is the predicted probability for class c observation o. Note that we ignore the unlabelled class in the loss computation. The weight term, $w_{c}$ is calculated as the inverse frequency of the annotation in the labelled dataset,

(7) $$w_{c}=\frac{\frac{1}{f_{c}}}{\sum_{i=1}^{Y_{N}}\frac{1}{f_{j}}},$$

where f is the frequency.

### 3.3 Performance evaluation metrics

We report the F1 over all folds to show the overall performance of the DL semantic segmentation model where no class is deemed more important. The F1 is defined as:

(8) $$\text{F1}=\frac{1}{Y_{N}}\sum_{i=1}^{Y_{N}}2\cdot \frac{\frac{{\mathrm{TP}}_{i}}{{\mathrm{TP}}_{i}+{\mathrm{FP}}_{i}}\times \frac{{\mathrm{TP}}_{i}}{{\mathrm{TP}}_{i}+{\mathrm{FN}}_{i}}}{\frac{{\mathrm{TP}}_{i}}{{\mathrm{TP}}_{i}+{\mathrm{FP}}_{i}}+\frac{{\mathrm{TP}}_{i}}{{\mathrm{TP}}_{i}+{\mathrm{FN}}_{i}}},$$

where $Y_{N}$ is the number of classes, TP is true positives, TN is true negatives, FP is false positives and FN is false negatives. Note that we can only report metrics for pixels which are labelled. We also report the accuracy (Acc):

(9) $$\mathrm{Acc}=\frac{\mathrm{TP}+\mathrm{TN}}{\mathrm{TP}+\mathrm{TN}+\mathrm{FP}+\mathrm{FN}},$$

and the true positive (TPR) and false positive rates (FPR):

(10) $$\mathrm{TPR}=\frac{\mathrm{TP}}{\mathrm{TP}+\mathrm{FN}},\mathrm{FPR}=\frac{\mathrm{FP}}{\mathrm{FP}+\mathrm{TN}}.$$

### 3.4 Data augmentations

To evaluate the effectiveness of the proposed *Calibration‐Jitter*, we compare it against several baseline pipelines. These include a no‐augmentation pipeline, a standard pipeline featuring geometric augmentations, and a bias pipeline that combines geometric and brightness bias augmentations. The brightness bias augmentation alters the image intensity randomly without accounting for spectral signatures and continuity, which can result in physically unrealistic outcomes. This technique is commonly applied to augment RGB images, which is why we have included it in our comparison. These pipelines serve as benchmarks for assessing the impact of the *Calibration‐Jitter* approach. For all the above pipelines the validation augmentations are:

- Resize to (256,256)
- Z‐score normalization

Note as a last step normalizing the image using z‐score normalization is often helpful, as it provides numerical stability for DL. The data $X_{\lambda }$ is normalized by the training fold's mean and standard deviations per channel,

(11) $${\text{Normalized}}_{\lambda }=\frac{X_{\lambda }-\mu_{\lambda }}{\sigma_{\lambda }},$$

where $\mu_{\lambda }$ is the mean pixel value of the dataset of each channel λ and $\sigma_{\lambda }$ corresponding standard deviation. For the no augmentation pipeline, in training we use the validation pipeline. The normal pipeline, in training, has augmentations:

- Resize to (310,280)
- Random crop to size (256,256)
- Random horizontal flip
- Random vertical flip
- Z‐score normalization

The bias pipeline, in training, has augmentations:

- Resize to (310,280)
- Random crop to size (256,256)
- Random horizontal flip
- Random vertical flip
- Brightness bias change (a coefficient drawn from the uniform distribution between −0.2 and 0.2 added to the original image)
- Z‐score normalization

The motivation behind the bias pipeline is to ensure the improved results are not simply due to regularization due to bias of spectra.

The *Calibration‐Jitter* pipeline follows the same augmentations but replaces the brightness bias augmentation with *Calibration‐Jitter*. In training, the augmentations are:

- *Calibration‐Jitter*
- Resize to (310,280)
- Random crop to size (256,256)
- Random horizontal flip
- Random vertical flip
- Z‐score normalization

To apply *Calibration‐Jitter*, first the standard deviation along the channel axis is computed, STD(λ), and is then multiplied by a coefficient drawn from a uniform distribution, U(−0.1,0.1). Finally this is added to the original white reference. The optimal choice for the parameters of the uniform distribution was selected trough an ablation experiment, shown in Table 1.

**TABLE 1:** Parameter study of coefficient (Coeff) used to control effect of *Calibration‐Jitter*.

Model	Coeff	F1 (%)
**UNet(ResNet34)**	U(−0.05,0.05)	72.04
	U(−0.1,0.1)	**73.04**
	U(−0.15,0.15)	71.39

Figure 3 shows an example image using *Calibration‐Jitter*. As illustrated, the output spectra are continuous, simulating different lighting conditions and thereby exposing the model to a diverse range of illumination variations during training. Notably, the pseudo RGB images clearly demonstrate that the lower bound makes the image darker, while the upper bound makes the image brighter.

### 3.5 Comparative analysis

Our proposed *Calibration‐Jitter* pipeline utilizes a randomly selected coefficient to determine the intensity of the augmentation relative to the original signal. To determine the optimal parameter, we tested various values, as detailed in Table 1. If the coefficient is too small, the augmentation's effect may be minimal, while if it is too large, it may overshadow the original signal. Our findings indicate that the most effective parameter range for our dataset is U(−0.1,0.1). For subsequent experiments, we used this coefficient range as the parameter.

The results with no augmentation—shown in Table 2 ‐ None—serve as a baseline to ensure that no augmentation performs worse than having none at all. By adding the Normal augmentations to the data pipeline we see a significant improvement in terms of results on all metrics for both Models UNet and SegFormer as expected.

**TABLE 2:** All metrics are the macro over classes. We report the: F1, accuracy (Acc), true positive rate (TPR) and the false positive rate (FPR).

Model	Augmentation	F1 (%) ↑	Acc (%) ↑	TPR (%) ↑	FPR (%) ↓
**UNet(ResNet34)**	None	59.55	87.54	58.91	9.38
	Normal	69.95	91.68	73.00	5.70
	Bias	71.53	92.10	73.40	5.49
	*Calibration‐Jitter*	**73.04**	**92.82**	**73.46**	**5.15**
**SegFormer**	None	68.79	90.00	72.21	6.17
	Normal	72.78	91.69	75.35	5.87
	Bias	72.32	91.72	76.03	**5.71**
	*Calibration‐Jitter*	**74.35**	**91.77**	**78.46**	**5.71**

The addition of the Bias augmentation further strengthens the results in terms of F1 using the UNet model (+1.58%) but also shows a reduction of F1 for the SegFormer model (−0.46%). However, the addition of Bias augmentation improves the accuracy, TPR, and FPR for both models, suggesting a regularization effect that enhances the models' learning.

In comparison to the proposed *Calibration‐Jitter* (as shown in Table 2), the Bias pipeline consistently performs worse in terms of F1 score (−2.03%), Accuracy (−0.05%), and TPR (−2.43%) for our top‐performing model, SegFormer, with similar trends observed for UNet. These results indicate that random intensity bias, which neglects spectral continuity, is less effective than our *Calibration‐Jitter* method, demonstrating the superiority of our approach.

We achieved the highest Macro F1 score of 74.35% with SegFormer using *Calibration‐Jitter*, compared to 73.04% with UNet. Unlike Bias augmentation, *Calibration‐Jitter* consistently improves on all metrics relative to Normal augmentation, underscoring the effectiveness of our method across various evaluation metrics.

Finally, we compare our best results using SegFormer with *Calibration‐Jitter* against the HIB benchmark base model. The benchmark achieves a best Macro F1 score of 70.2% using a neural network to classify each pixel, followed by clustering (spectral/spatial approach). Our method surpasses this benchmark, improving the F1‐macro score by approximately 4%. Qualitatively, *Calibration‐Jitter* also reduces the False Positive Rate (FPR) (as shown in Figure 4), leading to more localized tumor detection compared to the benchmark [24].

## 4 DISCUSSION

The aim of *Calibration‐Jitter* is to explicitly encourage the model to learn spectral features from illumination and sensor sensitivity changes, which are crucial for further deployment of the model in unseen hyperspectral data. These characteristics are often overlooked or underutilized in traditional approaches.

The results presented in Table 2 demonstrate the effectiveness of our proposed illumination augmentation technique across different evaluation metrics and segmentation model architectures. According to our 5‐fold cross‐validation, the models trained with *Calibration‐Jitter* consistently outperform in terms of F1‐score, accuracy, TPR, and FPR, for both the U‐Net and SegFormer architectures. This superiority highlights the invariance of the augmentation method to the choice of model architecture, underscoring its ability to enhance the generalization capabilities and performance of hyperspectral tissue segmentation models.

Qualitatively we can see the effect of *Calibration‐Jitter* in Figure 4. The augmented model produces more localized segmentation results and eliminates noise effects of isolated islands of incorrect predictions that were present in the non‐augmented model's output. The figures demonstrate that our proposed illumination augmentation technique, by simulating diverse lighting conditions through controlled offsets of the white reference, helps the model learn more robust features, leading to improved segmentation performance and mitigating overconfident predictions on unseen data.

Overall, the reported results and the parameter study provide strong evidence that our semantic segmentation approach with *Calibration‐Jitter* is a promising solution for this task. By explicitly enforcing the model to learn spectral features in different illuminations and sensor sensitivities, we have achieved superior performance compared to the only using geometric augmentations.

Recognizing the challenges of brightness augmentation in hyperspectral data, mainly due to the calibration step described in Figure 3, our method adjusts the intensity in a channel‐specific manner. We specifically designed *Calibration‐Jitter* to be straightforward, ensuring easy integration into existing data pipelines by only requiring the standard deviation calculation of the white reference. Although more sophisticated augmentations exist that could potentially yield superior outcomes, they often demand additional expertise in areas such as various light source spectra or tissue‐specific colour adjustments via semantic knowledge of the scenes. Despite its simplicity, our approach proves highly effective, which we believe is crucial.

## 5 CONCLUSION

In this paper, we have presented a novel augmentation technique. Our qualitative analysis illustrates the effectiveness of this augmentation method in regularizing overconfident predictions and removing small anomalies, leading to improved segmentation results. The quantitative results further corroborate the superiority of the *Calibration‐Jitter* augmentation. By achieving the highest macro F1‐score of 74.4%, we have surpassed the previously reported best performance on the HIB benchmark of 70.2%, obtained using a spectral/spatial approach. Overall, the findings presented in this work demonstrate the promising potential of our proposed augmentation method.

## AUTHOR CONTRIBUTIONS

**Alfie Roddan**: Conceptualization; data curation; formal analysis; investigation; methodology; project administration; resources; software; validation; visualization; writing—original draft; writing—review and editing. **Tobias Czempiel**: Methodology; writing—original draft; writing—review and editing. **Daniel S. Elson**: Supervision; writing—review and editing. **Stamatia Giannarou**: Funding acquisition; writing—review and editing.

## CONFLICT OF INTEREST STATEMENT

The authors declare no conflicts of interest.

## Data Availability

Research data beyond the contents of the paper are not shared.
